# A Research Journal Dedicated to the Effects of Xenobiotics in Organisms

**DOI:** 10.3390/jox10010003

**Published:** 2020-09-18

**Authors:** François Gagné

**Affiliations:** Aquatic Contaminant Research Division, Environment and Climate Change Canada, 105 McGill, Montreal, QC H2Y 2E7, Canada; Francois.gagne@canada.ca

The Journal of Xenobiotics (JoX), which was initially launched in January 2011 under PAGEPress (Italy) and recently with MDPI in September 2020, is devoted to the publication of novel and scientifically sound studies in the field of xenobiotics. Xenobiotics are broadly defined as chemicals foreign (strange) to life, i.e., are not produced in natural conditions. However, compounds of biological origins that are produced at scales not usually seen in nature could be considered as well. Hence, compounds observed at levels not usually found in natural conditions are considered as a special case of xenobiotics in the sense that abnormal levels of compounds even from biological origin could be considered unusual in organisms at high concentrations.

Natural products that are produced at a large scale for their beneficial properties (pharmaceuticals, foods, herbal medicine) and those that are inadvertently produced by human activities, such as polymers and toxins from algal blooms, are of interest for the journal. The scope of the JoX was developed to provide flexibility in order to address both the beneficial and harmful properties of new and existing compounds in various organisms.

The JoX promotes the rapid publication of peer-reviewed research articles dealing with either the pharmacological (beneficial) or toxicological (detrimental) properties of foreign compounds in all living beings. This dual approach should provide a more dynamic view on the health effects of xenobiotics of emerging interest in various ecosystems. Studies aimed at synthetizing products (e.g., products from green chemistry) or focusing on their release into the environment and understanding the basic/fundamental properties of xenobiotics that enable the development of biomarkers are welcome. Moreover, because organisms are rarely exposed to a single substance in the environment, studies dealing with the resulting toxic properties of complex mixtures, such as industrial or municipal wastewaters, or cocktails of pharmaceuticals threatening various health conditions, are also of value. For example, the interaction of pharmaceutical agents with nutrition status or diets, including herbal therapies, are of interest for the JoX.

The understanding of the combined effects of xenobiotics and their interaction with other chemicals is particularly of interest in the context of the 21st century. For example, the application of specialized materials derived from nanotechnology, which pervade many sectors of our economy, could also produce inadvertent health effects when released into the environment. A special case of nanomaterials concerns the release of plastic nanoparticles into the environment from the degradation of plastics. Nanoparticles could be readily absorbed in cells and produce unexpected biophysical changes at a cellular level. Indeed, plastic materials are found in very high levels (billions of tons) in the environment and degrade into smaller and smaller particles reaching the micro and nanoscale, which could pose a toxic risk in the long-term. Moreover, the use of drug vectors and environmental nanoparticles could bind existing contaminants (pesticides, polyaromatic hydrocarbons, etc.), influence their bioavailability and mitigate their effects.

In the context of global warming, more extreme episodes of drought and heavy rainfall could change their levels in the environment and modify organisms’ sensitivity to xenobiotics. Increased episodes of heavy rainfall could increase the release of untreated urban wastewaters (a complex cocktail of industrial and pharmaceutical products, among others) because of the volume limit of treatment plants to handle important volume changes. This further reinforces the need to better understand complex mixtures of xenobiotics. High throughput approaches from “omics” technologies should also contribute to an increase in our understanding on the beneficial/detrimental effects of newly produced xenobiotics or those of emerging interest. These approaches should provide critical information about the mechanisms of action and methods of identification of adverse outcome pathways.

The JoX is an open access journal, facilitating the accessibility of published articles among the scientific community, including research professionals and students, in the most efficient manner. The Editorial Board includes worldwide experts in different fields: natural and herbal medicinal products, drug therapeutics and biotransformation, clinical safety, occurrence and persistence of xenobiotics in the environment, nanotoxicology and ecotoxicology. The board members were handpicked to favor a multidisciplinary view of xenobiotics and were carefully selected across the planet to favor the best international perspective and audience for this open access journal.

The managing committee of this journal will ensure that all steps of submission, peer-review and final preparation of proofs respect the journal publication goals and deadlines. The journal also has an in-house English copyeditor to assist authors whose native language is not English. All this makes the JoX a convivial, simple and rapid platform for the publication of high-quality papers. The JoX welcomes short communications, Special Issue articles and communication from relevant sessions from international meetings dealing with the presence and the biological effects of xenobiotics. The JoX is a young and dynamic international journal that aims to reach the global scientific community as much as possible, for the benefit of us all. We are confident that this journal will contribute to the dissemination of peer-reviewed research data on either the beneficial or the harmful properties of chemicals from human activities in these times of global warming.

François Gagné, PhD

Editor in Chief for JoX.

## Short Bio of F Gagné


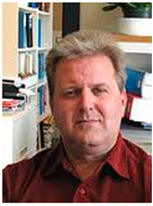


François Gagné is a Senior biochemical toxicologist at Environment and Climate Change Canada since 1994. He received his academic training in biochemistry and toxicology and earned his PhD (1996) in environmental toxicology at the University of Metz in France. He has published more than 250 papers (Scopus h index 46) in the area of biochemical ecotoxicology in international scientific journals, a book chapter and recently produced a book of methods in biochemical ecotoxicology (Elsevier Inc.). He is an associated professor at the Marine Science Institute at the University of Québec at Rimouski. His research interests reside in the area of toxicity of substances of emerging interest and the development of new approaches for risk assessment for new and exotic chemicals in the context of global warming. The use of non-linear approaches to study the oscillatory behavior and the fractal behavior of molecular changes in cells exposed to ultrafine plastic nanoparticles or other space-altering xenobiotics is currently of interest.

## History and Summary of MDPI

A pioneer in scholarly open access publishing, MDPI has supported academic communities since 1996. Based in Basel, Switzerland, MDPI has the mission to foster open scientific exchange in all forms, across all disciplines. Our 267 diverse, peer-reviewed, open access journals are supported by more than 67,200 academic editors. We serve scholars from around the world to ensure the latest research is freely available and all content is distributed under a Creative Commons Attribution License (CC BY).

